# A Herbal Formula in the Therapy of Acute Postviral Rhinosinusitis

**DOI:** 10.4274/tao.2020.6098

**Published:** 2021-03-26

**Authors:** Aleksandar Peric, Dejan Gacesa, Aleksandra Barac, Aneta Peric

**Affiliations:** 1 Department of Otorhinolaryngology, Military Medical Academy Faculty of Medicine, University of Defence, Belgrade, Serbia; 2 Ear Nose and Throat Hospital “Doctor Zutic”, Belgrade, Serbia; 3 Department of Infectious and Tropical Diseases, Belgrade University School of Medicine, Belgrade, Serbia; 4 Institute of Pharmacy, Military Medical Academy Faculty of Medicine, University of Defence, Belgrade, Serbia

**Keywords:** Rhinitis, herbal medicine, inflammation, nasal steroid, sinusitis, quality of life, endoscopy

## Abstract

**Objective:**

To assess the effects and adverse events of preparation Sinulan forte^®^ containing extracts of five medicinal plants in comparison to mometasone furoate nasal spray (MFNS) in therapy of acute postviral rhinosinusitis (APRS).

**Methods:**

We included 46 APRS patients in this prospective investigation and randomized to two groups. The patients in group 1 (n=23) received MFNS 200 μg two times/day for ten days, and patients in group 2 (n=23) received Sinulan forte^®^, tablets 225 mg per os, two times/day also for ten days. We evaluated the total symptom score (TSS), the separate scores for individual symptoms (nasal congestion, rhinorrhea, postnasal discharge, facial pain, impaired sense of smell), the quality-of-life outcome, and the findings from nasal endoscopy (edema of the nasal mucosa, nasal secretion) prior and after the therapy.

**Results:**

Significantly lower absolute post-treatment scores and better relative improvement were identified for TSS, nasal congestion, facial pain, loss of the sense of smell, edema of the mucosa and nasal secretion in patients receiving herbal preparation (group 2). However, lower absolute post-treatment score and better relative improvement were found for rhinorrhea and postnasal drip in group 1. Clinically important differences were found regarding the TSS and endoscopic findings, with no adverse effects in group 2, but in group 1 two patients had mild nasal bleeding and two had sensation of dryness in the nasal mucosa.

**Conclusion:**

Herbal product Sinulan forte^®^ can be a safe and effective treatment for APRS. Our results suggest no adverse events of this herbal preparation in comparison to intranasal corticosteroid spray therapy.

## Introduction

Acute rhinosinusitis (ARS) is an acute inflammatory disorder of the sino-nasal mucosa, and in more than 98% of the patients is caused by rhinoviruses, coronaviruses, influenza, and adenoviruses (1, 2, 3). This inflammation, usually known as common cold, can pass in 7-10 days with the appropriate symptomatic therapy. In some cases, however, it can be followed by acute postviral rhinosinusitis (APRS) with prolonged duration of nasal symptoms, requiring the use of medications (2, 3, 4). Inflammation caused by bacteria is found in only 0.5 to 2% of ARS patients, requiring antibiotic treatment (2, 3, 4). According to the current European guidelines for diagnostics and treatment of rhinosinusitis, there is a recommendation for use of intranasal corticosteroid sprays (INCS) and drops especially in the therapy of patients with APRS (5). A clinical trial conducted by Meltzer et al. (6) showed that the treatment of patients with uncomplicated ARS with mometasone furoate nasal spray (MFNS) was effective and relatively safe in a full dose of 400 µg daily. This is very important for the reduction of antibiotic prescriptions in these patients. However, there are evidenced problems with the use of INCS in patients with diabetes, enhanced intraocular pressure, cataract, arterial hypertension, and other disorders (6).

Herbal medicinal products have been used around the world for the treatment of many inflammatory diseases. In 2011, the World Health Organization estimated that 70–90% of the population of developing countries and almost 20% of the United States used herbal drugs, whereas in Europe these percentages are estimated to 10–20% (7, 8). *Andrographis paniculata* (creat or green chiretta) is an annual plant of the family *Acanthaceae,* native to South Asia, having antimicrobial and anti-inflammatory effects (9, 10). Several controlled clinical trials showed that this plant can be an effective and safe option in the treatment of uncomplicated upper airway inflammations (9, 10, 11, 12).Sinulan forte^®^ is a trademarked herbal preparation, available in tablets and composed of five herbal extracts: green chiretta (*Andrographis paniculata*, leaf), elder (*Sambucus* *nigra*, flower), common mullein (*Verbascum* *thapsus*, flower), European vervain (*Verbena officinalis*, herb), and gentian (*Gentiana lutea*, root). Herbal preparation with *Andrographis paniculata* extract is recommended in the European Position Paper on Rhinosinusitis and Nasal Polyps 2020 (EPOS 2020) for the treatment of common cold, but we found no randomized studies regarding the use of this herbal compound in the treatment of APRS (5). Our comparative study was designed to evaluate the effects and the safety of the herbal product Sinulan forte® and the intranasal corticosteroid MFNS in the treatment of patients with APRS.

## Methods

### Study Design

This was a randomized, prospective, open-label and non-inferiority study on the treatment of APRS. The study was conducted according to the Helsinki Declaration, from January through December 2019 in our Department of Otorhinolaryngology. The protocol for investigation is approved by the Ethics Committee of the Military Medical Academy, Belgrade, Serbia (MMA no: 05/2019). Written informed consent was obtained from every patient.

### Inclusion and Exclusion Criteria

Forty-six subjects with APRS were recruited for participation in this study. In line with the EPOS 2012 (13), patients had inflammatory changes in the nasal cavity and sinuses, increased symptoms (nasal obstruction/congestion, rhinorrhea, postnasal drip, facial pain/pressure, and loss of the sense of smell) after five days or persistent complaints after 10 days, but no longer than 12 weeks. Endoscopic examination of the patients showed edematous nasal mucosa and increased middle meatal secretion.

Criteria for exclusion were: patients aged <18 and >65 years, having chronic rhinosinusitis (CRS), previous surgery in the sinonasal region, deformation of the nasal septum and turbinate hypertrophy that significantly disrupt the nasal airflow and INCS application, different systemic disorders that can affect the nose and sinuses (non-eosinophilic and eosinophilic granulomatosis with polyangiitis, Kartagener’s syndrome, cystic fibrosis, etc.), immunologically associated diseases (multiple sclerosis, polyarthritis, rheumatoid arthritis, diabetes mellitus), sensitivity to non-steroid anti-inflammatory drugs, seasonal allergic rhinitis, bronchial asthma, allergic reactions to medicines used in this study. Also, patients who had used oral or topical antihistamines, corticosteroids, and antibiotics a month prior to the study and patients who used sympathomimetics and mucolytics within the seven days before the study were also excluded. Current cigarette smoking, pregnancy and lactation were also criteria for exclusion. Patients who had complaints of common cold within five days resolution and severe bacterial ARS, with fever higher than 38 °C, severe facial pain and mucopurulent discharge with unilateral predomination were excluded. To exclude the patients with bacterial ARS, aspirate from the middle meatus was taken from every patient and samples were cultivated on blood agar (HiMedia™ Laboratories, Mumbai, India) for pathogenic bacteria.

### Randomization

**Figure 1:**
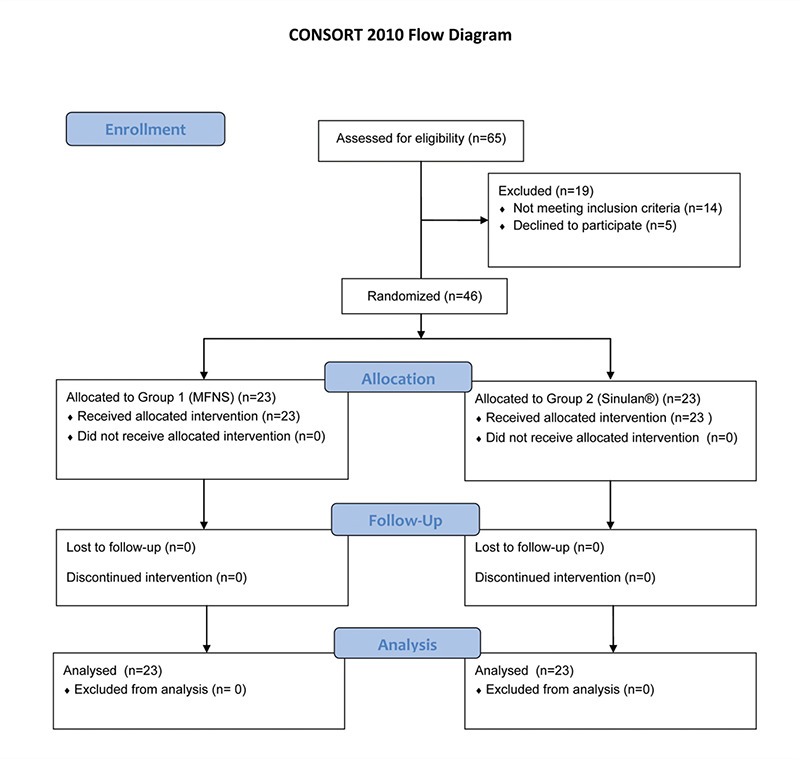
Randomization was performed in accordance with the CONSORT statement. Sixty-five patients (n=65) diagnosed with APRS were found eligible for the study. Five (n=5) patients refused to participate and fourteen (n=14) did not meet the inclusion criteria. Forty-six (n=46) patients were thus enrolled and randomized to groups 1 (n=23) and 2 (n=23) CONSORT: Consolidated Standards of Reporting Trials, APRS: Acute postviral rhinosinusitis, n: Number

The patients were randomly divided according to the CONSORT statement. Sixty-five subjects with diagnosis of APRS were recruited for this study. Five patients did not accept to participate, and fourteen subjects did not meet the inclusion criteria. Finally, forty-six (n=46) patients were selected and assigned to group 1 (n=23) and group 2 (n=23). Computer-generated random allocation was used for assignment of patients into two groups. The participants were deemed eligible by the researcher. The researcher then informed the nurse about the eligibility and she assigned the participants to group 1 or 2. Figure 1 presents the study profile.

### Treatment

Group 1 (n=23) received MFNS (Mometazon Sandoz®, Lek Pharmaceuticals D.D., Verovskova 57, 1526 Ljubljana, Slovenia) 200 µg two times daily (two sprays in both nostrils in the morning and in the evening) for 10 days. These patients were informed about the correct application of INCS. Group 2 (n=23) received herbal preparation Sinulan forte® 225 mg tablets (Walmark, A.S., Oldrichovice 44, 73961 Trinec, Czech Republic), two times daily for 10 days. The drugs were provided to the patients after randomization. Both the researchers and patients knew which treatment was being administered.

### Clinical Evaluation

Levels of ARS symptoms were evaluated at the start of the study (visit 1) and within two days after the end of therapy (visit 2) using a 10 cm visual analogue scale (VAS) (0–10 cm; from 0=absence of symptom to 10=maximum symptom intensity). The use of VAS was applied and explained to the patients by the nurse after the randomization. Patients self-reported the intensity of their symptoms. Symptoms scored from 0 to 3 were indicated as “mild ARS;” symptoms scored from 4 to 7 were indicated as “moderate ARS.” Participants with “severe ARS,” i.e., scored from 8 to 10 and reported fever higher than 38 °C were excluded. The participants self-assessed the intensity of their symptoms and noted the therapy use on diary cards, two times daily, after the taking their medication. At visit 2, the researcher recorded the scores and evaluated the patients’ treatment compliance based on their diary cards. The health-related quality of life (QoL) score was evaluated at visits 1 and 2 with the Sino-Nasal Outcome Test 20 (SNOT-20). This 20-item questionnaire can be used for the assessment of social and emotional consequences of ARS symptoms. At visits 1 and 2, two independent rhinologists used a 4 mm 0° endoscope (Storz SE & Company, Tuttlingen, Germany) to evaluate the presence of mucosal edema and nasal secretion in the middle meatus on the same patient. A four-point scale described by Pfaar et al. (14) was used for the evaluation of mucosal edema and nasal secretion. Presence of edema was scored from “no edema” (0) to “severe edema” (3); nasal secretion from “none” (0) to “profuse” (3). The maximum bilateral score for separate endoscopic sign is 6. Following the EPOS 2012 guideline (13), we did not use radiological examinations (X-ray, computed tomography, magnetic resonance imaging) for the diagnosis and the evaluation of the treatment outcomes.

The efficacy endpoints were mean total symptom score (TSS); sum of the scores for 5 symptoms, individual symptom scores, scores for endoscopic findings (edema, nasal secretion), and SNOT-20 score at the visit 1 and visit 2.

### Follow-up

All patients were asked to come for the follow-up visit on days 10^th^ and 20^th^, and TSS and endoscopic parameters were evaluated due to the potential risk of recurrence.

### Safety

Reported adverse effects were noted during the study, including the follow-up period, by grading their severity as mild, moderate, and severe. At visit 2, we performed laboratory tests, evaluation of vital signs and nasal examination (rhinoscopy, endoscopy). The participants were informed about the possible adverse effects of both medications. All possible complications of severe form of ARS (orbital, intracranial or bony) were noted during the study.

### Sample Size Calculation

Calculation of sample size was based on a requirement to reach the minimal differences in TSS and endoscopic findings between the two treatment options. According to Meltzer et al. (15), the calculation of minimal clinically important difference (MCID) is based on a proposal provided by the Agency for Healthcare Research and Quality. This distribution-based method relied on the statistical distributions of data concerning the TSS and endoscopic findings. The calculation is performed by the formula: MCID=0.5 (50%) of the sum of pre-treatment standard deviations (SDs) of TSS/endoscopic signs in both treatment groups. The sufficient total sample sizes providing at least 80% power of study, at level of significance of 0.05 to reach a MCID in TSS of 1 and endoscopic score of 1 were 23 participants in each group.

### Statistical Analysis

The parameters were expressed as mean ± SD. For between-group comparison, we used the non-parametric Mann-Whitney U test. For paired comparisons within a group, we used the Wilcoxon’s test. To calculate the relative improvement of each parameter weighed with the pretherapeutic value, we used the formula: post-therapeutic value - pretherapeutic value/pretherapeutic value * 100. The statistical significance (p) is set at the level of 0.05. The analysis was performed using the version 15.0 of the Statistical Package for the Social Sciences (SPSS) software (SPSS Inc., Chicago, USA).

## Results

### Demographic Data

Forty-six participants (27 males and 19 females) aged 19 to 63 years (mean age: 41.37±32.65) suffering from APRS were enrolled in the study. Demographic characteristics of participants are presented in Table 1.

### Clinical Data

Data presenting pre- and post-treatment TSS, individual scores for each nasal symptom, SNOT-20 and endoscopic findings are presented in Table 2. Results related to all parameters’ relative changes after the two different therapies are presented in Table 3.

At the start of study (visit 1), we found no differences regarding the TSS and individual symptom scores between the two treatment groups. We also found no differences between the treatment groups regarding SNOT-20, mucosal edema, and nasal secretion (Table 2).

After the treatment, we found a significant decrease in all parameters, including a SNOT-20 score in both groups of patients.

At visit 2, comparing the post-treatment levels of parameters, we observed lower TSS and lower scores for nasal obstruction/congestion, facial pain/pressure, impaired sense of smell, as well as endoscopically assessed mucosal edema and nasal secretion in group 2. We did find, however, lower post-treatment rhinorrhea and postnasal drip scores in APRS patients treated with MFNS (group 1) compared to those treated with herbal preparation (group 2). However, we found no post-treatment differences in SNOT-20 between the two groups (Table 2).

**Figure 2:**
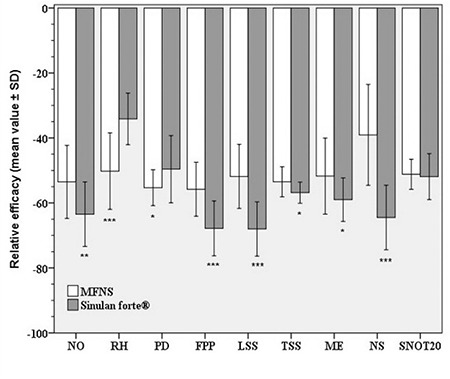
Differences in the relative improvement of the clinical parameters after the treatment with two different preparations. NO: Nasal obstruction, RH: Rhinorrhea, PD: Postnasal drip, FPP: Facial pain/pressure, LSS: Loss of the sense of smell, ME: Mucosal edema, NS: Nasal secretion, TSS: Total symptom score, SNOT20: Sino-nasal outcome test 20. ***p<0.001 vs corresponding group, **p<0.01 vs corresponding group, *p<0.05 vs corresponding group

When we compared the relative improvement for each parameter, we found significantly higher relative improvement for TSS, nasal obstruction/congestion, facial pain/pressure, loss of sense of smell, and for mucosal edema and nasal secretion in group 2. However, relative improvement for rhinorrhea and postnasal drip score was better in group 1. We found no differences in SNOT-20 relative improvement between two groups (Table 3) (Figure 2).

We found that the MCID for TSS between the two groups after the treatment should be 1.23. The difference between post-treatment values was: 15.17–13.3=1.87. As our difference was higher than 1.23, this meant clinically important difference in TSS. We also found that the MCID for the sum of endoscopic findings between two groups should be 0.53. The difference between post-treatment values in endoscopic findings was: 5.57–3.96=1.61. As our difference is much higher than 0.53, this, too, meant clinically important difference in endoscopic findings.

### Follow-up

All patients were proceeded to the follow-up for the evaluation of TSS, mucosal edema and nasal secretion. As presented in Table 4, we found no important changes in the trends of the differences between the two treatment groups on day 10^th^ and day 20^th^ following visit 2.

### Safety

The patients from the herbal preparation group (group 2) did not report any adverse effects, whereas from the MFNS group (group 1) two patients reported mild epistaxis and two reported mild dryness sensation of the nasal mucosa. No participants were found with disturbed vital signs and laboratory findings.

## Discussion

This is the first randomized study comparing the efficacy and safety of an herbal preparation with dry extracts of five medicinal plants with *Andrographis paniculata* extract as a main constituent to INCS treatment on nasal symptoms, endoscopic findings and QoL in patients with APRS. The result demonstrated that both the herbal medicinal product and INCS reduced all symptoms and endoscopic signs and improved QoL in patients with APRS. However, our results suggest slightly better relative improvement in all clinical parameters, except for rhinorrhea and postnasal drip scores, in patients treated with Sinulan forte^®^ than in those treated with MFNS. Finally, our results showed clinically important post-treatment differences in both TSS and endoscopic findings between the groups.

Herbal medicinal products, which contain extract of green chiretta (*Andrographis paniculata*) as their main compound are reported to improve the symptoms in patients with common cold (9, 10, 11, 12). The main active ingredient of *Andrographis paniculata* leaf has not been fully identified, but it is generally assumed to be andrographolide. This lactone has strong effects against viruses, and acts against *Streptococcus pneumoniae, Staphylococcus aureus* and *Escherichia coli *(16). Andrographolide has strong anti-inflammatory effects. It inhibits lipopolysaccharide-stimulated nitric oxide (NO) and pro-inflammatory cytokine production in the inflamed tissue of the nasal mucosa (16). Andrographolide significantly inhibits interleukin-6 and interleukin-17 production in monocytes isolated from CRS patients with nasal polyp (17).

However, other constituents in Sinulan forte^®^ also show strong antiviral and anti-inflammatory activities. The flower of common mullein has many bioactive substances (glycosides, saponins and terpenoids) that exhibit strong anti-influenza virus activity, as well as anti-*Staphylococcus aureus*, antioxidant, and wound-healing activity (18). European vervain, gentian and elder have anti-inflammatory effect that can be attributed to the antiviral effect of bioflavonoids, especially on rhinoviruses, adenoviruses, corona viruses, coxsackie and influenza virus (19).Bioflavonoids inhibit neuraminidase, an enzyme of great importance for replication of viruses (19). These three plants also have bacteriostatic action (19).These anti-inflammatory, antiviral, and antibacterial effects of Sinulan forte^®^ constituents results to a stronger improvement of nasal obstruction/congestion, rhinorrhea, facial pain/pressure, and loss of sense of smell in comparison to patients treated with MFNS monotherapy. The better resolution of endoscopically evaluated nasal secretion from the middle meatus in our patients treated with the herbal preparation can also be explained by the antiviral and bacteriostatic actions of these constituents (19).

Following the treatment, at visit 2, the patients treated with MFNS had lower scores for rhinorrhea and postnasal discharge, and better relative improvement in rhinorrhea and postnasal drip scores than the patients treated with herbal preparation. These findings are not in accordance with the lower nasal secretion scores and better relative improvement in nasal secretion scores seen in in group 2 following the treatment. We could explain this unusual feature with the stimulative effect of European vervain, gentian and elder have on mucociliary clearance (20). ARS is characterized by disturbance in mucociliary clearance caused by infection and inflammatory changes in the nasal mucosa. This mucosal clearance depends on the active transport of chloride ions (Cl-) through the respiratory epithelium, strongly regulated by bioflavonoids, the main pharmacological constituents in European vervain, gentian and elder. This increased transepithelial transport results in better hydration of the mucus and reduction of its viscosity (20). So, three constituents from Sinulan forte^®^ stimulate mucociliary clearance, resulting in higher rhinorrhea and postnasal discharge scores in these patients. Therefore, intranasal corticosteroids have an anti-inflammatory action, resulting in the inhibition of secretion from the mucosal glands (21). So, the patients treated with the herbal product have higher scores of rhinorrhea and postnasal discharge after the treatment compared to those treated with MFNS.

Although majority of the studies recommend intranasal corticosteroid therapy for 14-15 days, there is no clear recommendation in the European (EPOS 2012) or the American (ICAR 2016) guidelines regarding the duration of intranasal corticosteroid therapy in patients with ARS, particularly in those suffering from APRS (3, 6, 13, 22, 23). The duration and nature of APRS symptoms were the main reasons why we decided to treat our patients for 10 days. First, we wanted to coordinate the duration of treatment with Sinulan forte^®^ with the duration of the MFNS treatment. Our results showed no compromise in the efficacy of MFNS as the patients had very good improvement in terms of clinical parameters. However, our results suggest that oral therapy with the herbal preparation have slightly better clinical efficacy than topical corticosteroid therapy. As APRS has more persistent symptoms than common cold, the duration of the herbal treatment was prolonged. In a study by Saxena et al. (11), the patients with common cold were given the preparation with *Andrographis paniculata* (KalmCold™) two times daily for 5 days, whereas the patients in our study were treated for 10 days. The patients in group 1 from our study were given 200 µg of MFNS twice daily for 10 days, 400 µg daily in total. According to the study by Meltzer et al. (6), MFNS 200 µg once daily and twice daily (400 µg) for 15 days was significantly superior to placebo in the treatment of patients with mild-to-moderate ARS. Finally, from a safe standpoint, our results of the 20-day follow-up after the end of medication use could be an apology for our choice of 10-day treatment. The results showed that there appeared to be no greater risk of recurrence of bigger exacerbation of APRS symptoms after MFNS treatment compared to Sinulan forte^®^ treatment.

According to our results, treatment by herbal preparation and INCS almost equally reduces the SNOT-20 score. We found no post-treatment absolute differences and no difference in relative improvement in QoL between the two groups. However, TSS and individual symptom scores were significantly lower after the treatment with herbal drugs than with MFNS. VAS is a psychometric measurement instrument to subjectively quantify patients’ symptom severity. The SNOT-20 is a questionnaire designed for the assessment of QoL in patients with rhinosinusitis (24). According to papers published in the past ten years, correlation between VAS and QoL tests (SNOT-20, SNOT-22) is well established for patients with CRS (25). However, we found no papers concerning such correlation in patients with ARS, especially in those with APRS. One possible conclusion could be that in patients with APRS, improvement in nasal symptoms assessed with VAS does not follow the improvement in the QoL, estimated by SNOT-20.

While previous studies reported no serious adverse effects of *Andrographis paniculata* and other herbal constituents of Sinulan forte^®^, the minor adverse events reported were mainly gastrointestinal, such as nausea and diarrhea, and allergic reactions to the preparation constituents (9, 10, 11, 12). We encountered no adverse effects in our patients that were treated with the herbal preparation, whereas in the group treated with MFNS two patients reported mild nasal bleeding and two reported mild sensation of dryness in the nasal cavity. As previously noted, INCS decrease the activity of glands situated in the nasal mucosa (21). The most common local adverse effects of INCS use include epistaxis and nasal dryness (26). Rate of epistaxis has been reported to be about 5% in patients treated with INCS. According to a recent systematic review of the literature, nasal bleeding can be a result of small mechanical trauma of the nasal mucosa by nasal applicator tip of the INCS device against the nasal septum rather than a result of mucosal atrophy (26).

The presented study has limitations, since it was not a multi-center study and the sample size was relatively small. The evaluation of nasal symptoms was dependent on the subjective sensation of the patients. On the other hand, we used endoscopic examination for the objective assessment of the local findings in the nasal cavity. Although randomized and prospective, our study was conducted as an open label study. Evidence for the clinical effects of herbal medicines in the treatment of common cold and APRS is limited in the medical literature and only several placebo-controlled studies investigating the efficacy of *Pelargonium sidoides* (EPs^®^ 7630), five herbal compounds (BNO 1016), Myrtol standardized (GeloMyrtol^®^), and *Cyclamen europaeum* (Nasodren^®^) reported benefit of treatment versus placebo, with significantly reduced severity and duration of disease, and without serious side-effects (27, 28, 29, 30). So, there is a need for further placebo-controlled studies that can provide better evidence of the effects of Sinulan forte^®^ and other herbal preparations in the treatment of APRS.

## Conclusion

Our results demonstrated statistically significant and clinically relevant improvement in TSS after the treatment with the herbal product Sinulan forte^®^ compared to MFNS. Further, the herbal preparation had better effects on nasal congestion, facial pain, and loss of the sense of smell, and on endoscopic signs in patients with APRS compared to MFNS. We found no adverse effects in patients treated with herbal preparation, suggesting that this treatment can be safer compared to INCS treatment in patients with this uncomplicated form of ARS.

**Main Points**

• Herbal preparation with Andrographis paniculata extract is recommended in the current European guidelines for treating common cold, but we found no studies regarding the use of this herbal compound in the treatment of acute postviral rhinosinusitis (APRS).

• The aim of our study was to evaluate the efficacy and safety of the combined herbal medicinal product Sinulan forte^®^, which contains the extracts of five medicinal plants with *Andrographis paniculata* as a main constituent, in comparison to mometasone furoate nasal spray (MFNS) when treating the patients with mild to moderate APRS.

• Our results showed statistically significant and clinically relevant improvement in the total symptom scores (TSS) of the patients treated with the herbal product compared to those of the patients treated with MFNS.

• The herbal preparation has better effects on nasal obstruction, facial pain/pressure, and impaired sense of smell, as well as on endoscopic findings in comparison to MFNS.

• No adverse events were encountered in patients treated with herbal preparation, suggesting that this treatment can be a safe treatment option in patients with APRS.

## Figures and Tables

**Table 1:**
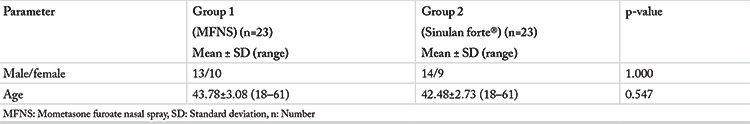
Demographic characteristics of the study population

**Table 2:**
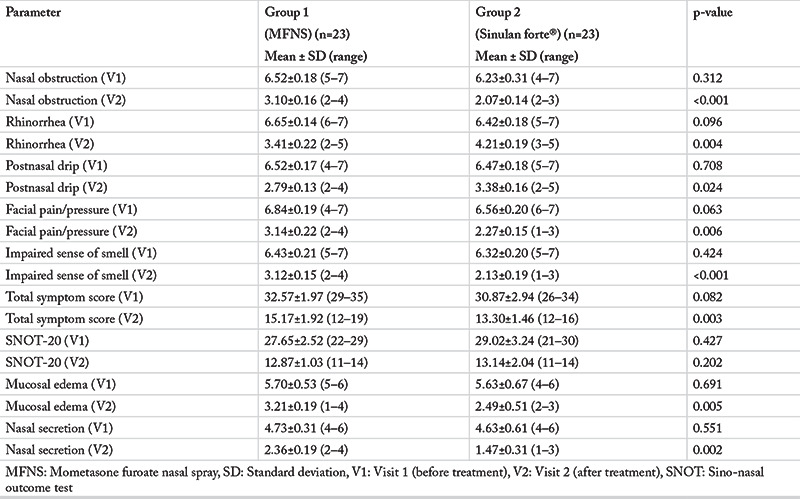
Clinical parameters before and after treatment

**Table 3:**
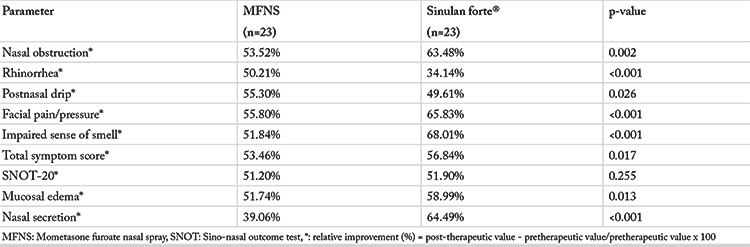
Differences in the relative improvement of the clinical parameters after the treatment with two different preparations

**Table 4:**
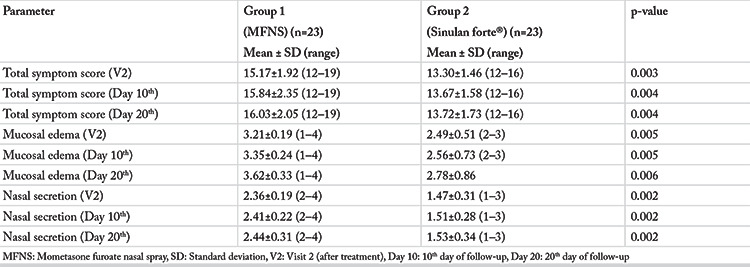
The main clinical parameters during the follow-up
